# Discovering antiviral restriction factors and pathways using genetic screens

**DOI:** 10.1099/jgv.0.001603

**Published:** 2021-05-21

**Authors:** Chloe E. Jones, Wenfang S. Tan, Finn Grey, David J. Hughes

**Affiliations:** ^1^​ Biomedical Sciences Research Complex, School of Biology, University of St Andrews, St Andrews, KY16 9ST, UK; ^2^​ Division of Infection and Immunity, The Roslin Institute, University of Edinburgh, Edinburgh, EH25 9RG, UK

**Keywords:** Innate immunity, CRISPR/Cas9, genome-wide screens, RNAi, antiviral immunity, interferon

## Abstract

Viral infections activate the powerful interferon (IFN) response that induces the expression of several hundred IFN stimulated genes (ISGs). The principal role of this extensive response is to create an unfavourable environment for virus replication and to limit spread; however, untangling the biological consequences of this large response is complicated. In addition to a seemingly high degree of redundancy, several ISGs are usually required in combination to limit infection as individual ISGs often have low to moderate antiviral activity. Furthermore, what ISG or combination of ISGs are antiviral for a given virus is usually not known. For these reasons, and since the function(s) of many ISGs remains unexplored, genome-wide approaches are well placed to investigate what aspects of this response result in an appropriate, virus-specific phenotype. This review discusses the advances screening approaches have provided for the study of host defence mechanisms, including clustered regularly interspaced short palindromic repeats/CRISPR associated protein 9 (CRISPR/Cas9), ISG expression libraries and RNA interference (RNAi) technologies.

## Introduction

As obligate intracellular pathogens, viruses rely on host machinery to complete their replicative cycle; virus–host interactions are therefore crucial to the infectivity of the virus particle and its ability to cause disease [1]. Whilst there are host dependency factors present in cells that permit the replication of viruses, there are also antiviral factors that restrict the pathogen’s ability to replicate [2]. Despite many viruses encoding countermeasures to these antiviral factors [3], our understanding of them is pivotal to understanding their weaknesses and aids our understanding of how infection can be controlled.

Upon encountering a pathogen, the innate immune response is activated and subsequently triggers adaptive immunity. A component of the innate immune response, the evolutionarily conserved interferon (IFN) system [4], has well-established antiviral defence roles. This response slows viral replication and the rate at which infection spreads before activation of the adaptive immune response [5, 6]. Furthermore, IFNs are important immunomodulatory cytokines that regulate the magnitude of the host response and therefore limit tissue damage [7].

All cells express pathogen recognition receptors (PRRs) that upon recognition of pathogen-associated molecular patterns (PAMPs), generally consisting of viral nucleic acids upon infection with viral pathogens, establish a signalling cascade leading to the production of cytokines, including IFN [8]. There are three families of IFN, type I, II and III. Whilst type II IFN (IFN-γ) regulates the cell-mediated response to infection, type I IFN, consisting of 13 subtypes including IFN-α and IFN-β, and type III IFN (IFN-λ) establish an antiviral state in both the infected cell and surrounding, non-infected cells [7, 9, 10]. Secreted IFN binds to cell surface receptors and initiates a canonical signalling cascade dependent on the Janus kinase (JAK)–signal transducer and activator of transcription (STAT) pathway. Activation of the JAK–STAT pathway results in the production of hundreds of interferon stimulated genes (ISGs) culminating in extensive biological effects (reviewed by Fensterl *et al.* [6]).

Untangling the biological consequences of this large physiological response is complicated. Many ISGs have broad antiviral activity, such as double-stranded RNA (dsRNA)-dependent protein kinase R (PKR) that induces a shutdown of general protein translation upon activation and phosphorylation of eukaryotic translation initiation factor 2A (eIF2A), and the 2′,5′-oligoadenylate synthetase (OAS)–RNase L pathway that degrades single-stranded RNAs (ssRNAs), including mRNAs of viral and cellular origin [11, 12]. Others, such as IFN-induced protein with tetratricopeptide repeats (IFIT) and IFN-induced transmembrane (IFITM) proteins inhibit specific viruses, but many viruses remain insensitive to them. Because IFN signalling causes an extensive physiological reaction, many ISGs play critical regulatory roles that temper the response and prevent autoinflammatory disease and excessive tissue damage. For these reasons, and since the function(s) of many ISGs remains unexplored, genome-wide approaches are well placed to investigate which aspects of this broad response results in an appropriate, virus-specific antiviral phenotype.

### Advances in molecular tools enabling genome-wide approaches

Advances in molecular tools have transformed our ability to investigate virus–host interactions and facilitated the use of genome-wide genetic screening in the field. The development of next-generation sequencing (NGS) and genome editing tools have enabled sequence-based analysis with improved output reliability [13, 14]. These tools have not only allowed for specific targeting of genes but make anticipated phenotypic expression more easily interpretable [13, 15]. Due to the nature of genetic screening, the ability to reliably characterise genes that produce the desired output is paramount.

**Fig. 1.**
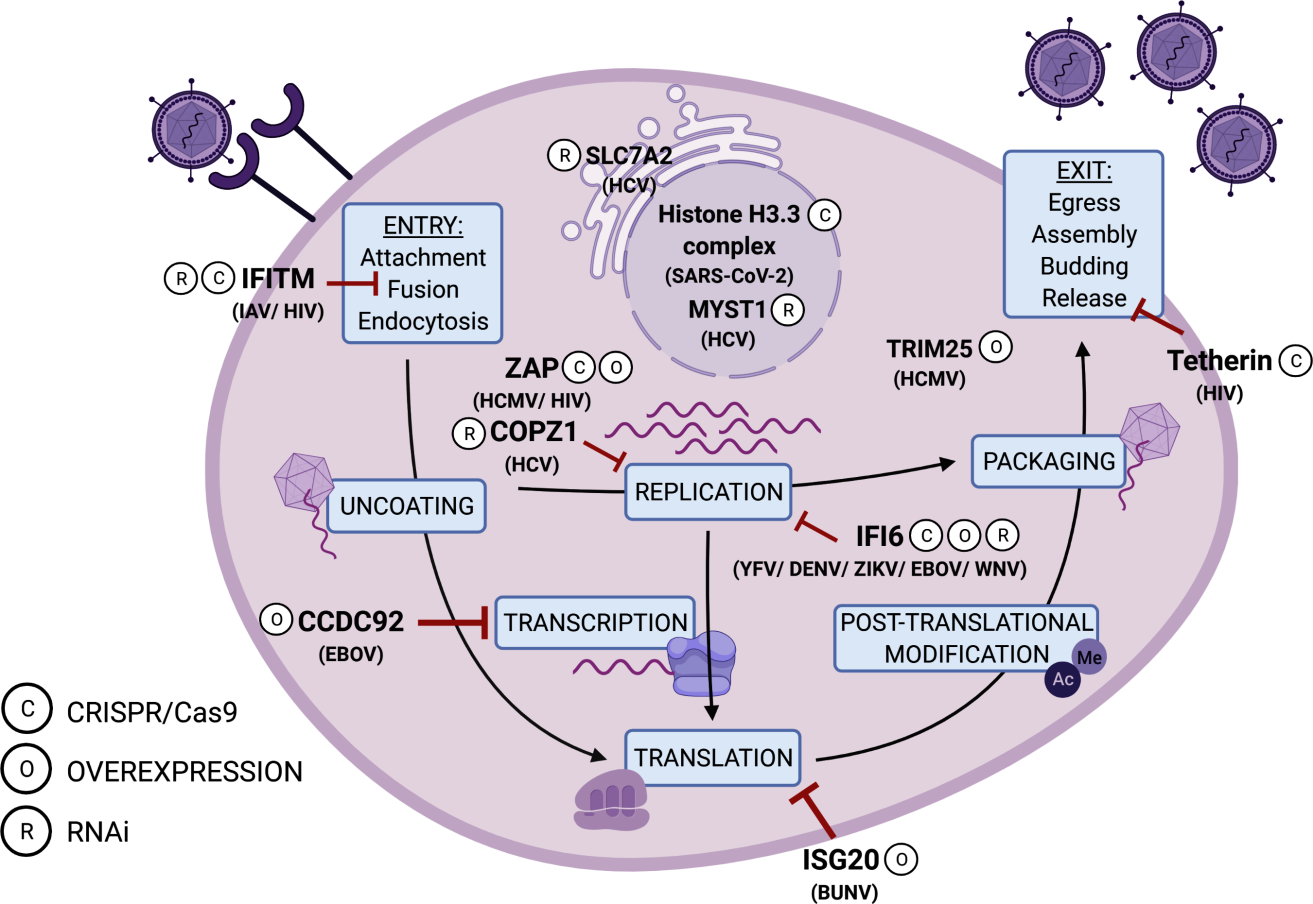
Antiviral factors identified using screening methods. A schematic illustrating a selection of antiviral factors identified during genome-wide screening. Each antiviral factor is attributed to a stage in the viral life cycle corresponding to literature references throughout this review [52, 53, 63, 65, 69, 85, 87, 141]; it should be noted that many ISGs have been seen to act at additional stages. The key indicates the screening type used to identify each example: (**c**), clustered regularly interspaced short palindromic repeats/CRISPR associated protein 9 (CRISPR/Cas9), (**o**), Overexpression, (**r**), RNA interference (RNAi). Abbreviations used: HCV; Hepatitis C Virus, SARS-CoV-2; Severe Acute Respiratory Syndrome Coronavirus 2, IAV; Influenza A Virus, HIV; Human Immunodeficiency Virus, HCMV; Human Cytomegalovirus, EBOV; Ebola Virus, YFV; Yellow Fever Virus, DENV; Dengue Virus, ZIKV; Zika Virus, WNV; West Nile Virus, BUNV; Bunyavirus. Created with biorender.com biorender.com.

Whilst previous studies investigating virus–host interactions have been limited to investigating a single to a few genes at a time, screening platforms are a high-throughput method allowing for the identification of many genes. This lends itself to the study of broad signalling systems, such as the IFN response, by identifying complex network interactions that work in concert. Genome-wide genetic screening has widely been used to investigate virus–host interactions, as will be further discussed, and up to the date of publication, many of these screens have identified host dependency factors in the cell required for successful virus replication [16–21]. These screens have also been used to identify antiviral factors (Fig. 1).

As mentioned, the IFN response results in the production of hundreds of ISGs [6]. This is a largely redundant response as it is expected that only a small number of these proteins will significantly contribute to the antiviral response. Instead, there are many ISGs which have minor effects on viral replication resulting in a synergistic effect. However, which of the hundreds of ISGs are antiviral for which virus is mostly unknown and finding the associated restriction factor could be likened to finding a needle in a haystack. Results from previous work have indicated that mice defective in a broad-ranging ISG, such as PKR or OAS, are still able to produce an antiviral response indicating that several factors are likely to be at play [2, 22]. The question then becomes; if several ISGs each play a modest role [23], how are functional components of this vast redundant response identified without an overarching phenotype? Genome-wide genetic screening has the ability to identify novel genes and exclude genes not involved in the antiviral response, due to its high-throughput nature and size of gene libraries available, therefore aiding the identification of redundant genes in the response. Subsequent comparative analysis of validated genes can identify whole pathways involved in virus–host interactions; something not possible in single-gene studies [24, 25].

### Screening platform design

Screens, either RNA interference (RNAi), clustered regularly interspaced short palindromic repeats/CRISPR associated protein 9 (CRISPR/Cas9) or overexpression, are performed in an arrayed or pooled format in which the genetic perturbators are arranged individually in multi-well plates or are cloned to create a library respectively. Each screening platform has advantages and disadvantages. Arrayed libraries are preferred for experimental designs investigating a number of phenotypes or with small culture volumes [13]. They permit plate reader screening [26, 27], allowing individual wells to be treated with drug compounds, infected with a pathogen or put under environmental stress. Conversely, pooled screening allows for quantification of constructs within a population whilst being less expensive and labour intensive [13]. It is also possible to split pools into several smaller pools allowing for the comparison of two or more populations by microarray or NGS.

There are three primary readouts of screens; multi-well plate readers [26, 27], microscopy [28] or flow cytometry [29, 30]. A problem however with using microscopy to investigate virus-host interactions is the variability in results. Previous studies have yielded varying results dependent on if a host cell component indicative of infection is imaged or if infection is monitored using a reporter virus, such as GFP [31].

## CRISPR/Cas9 Screening

Genome-wide CRISPR/Cas9 knockout screening utilises clustered regularly interspaced short palindromic repeats (CRISPR)/CRISPR associated protein 9 (Cas9) genome editing; first described to target and cleave the human genome in 2013 to introduce loss-of-function mutations [32–35]. In CRISPR/Cas9 loss-of-function genetic engineering, the Cas9 enzyme is programmed with a single guide RNA (sgRNA) complementary to the gene of interest and upon complementary binding, site specific DNA cleavage results in a double strand break (DSB). In the absence of a homologous sequence, the DSB is repaired via non-homologous end joining (NHEJ); an error-prone process resulting in insertions and deletions (INDELS). This mechanism is exploited in genome engineering as the resulting frameshift mutations disrupt gene function (reviewed elsewhere [36, 37]). CRISPR/Cas9 screening utilises this technology in a high-throughput manner to provide an experimental design that works in a phenotype-to-genotype direction [13, 15]. This forward screening approach allows the identification of unexpected genes involved in virus-host interactions that are unlikely to be identified through hypothesis-based reverse genetic screening approaches.

Pooled screening introduces a library of sgRNAs either alongside Cas9 or into Cas9-expressing cells primarily via lentiviral transduction at a low multiplicity of infection (MOI) in order to reduce multiple vector uptake by cells; other delivery methods include PiggyBac transposons [38] and adeno-associated virus (AAV) delivery [39]. This results in permanent modification and complete knockout of the targeted gene. A selection pressure is then applied to select for successfully transduced cells and an assay is required to separate cells with the phenotype of interest. Genomic DNA is harvested and sgRNA isolated and amplified for NGS. Enrichment significance is then calculated for each sgRNA isolated (detailed protocols have been published previously [40, 41]).

### Alternative strategies in CRISPR/Cas9 screening experimental design

Pooled screens can be negative or positive screens. Negative screens work through performing NGS at both the beginning and end of applying the selection pressure and identifying sgRNAs that have been depleted; these targeted genes are therefore critical to cell proliferation and survival. Conversely, positive selection screens require only a single round of NGS. However, it should be noted that most protocols involve sequencing at the beginning and end to control for factors such as enrichment that occur prior to infection with the pathogen of interest. Following application of the selection pressure, cells that have survived are sequenced and enriched sgRNAs correspond to target genes whose knockout leads to cell survival or that ordinarily restrict cell growth, such as tumour suppressor genes. Screens can also be performed with a reporter-based assay. These are followed by fluorescence activated cell sorting (FACS) enrichment of cells expressing sgRNAs rather than enrichment by cell growth or viability [42, 43]. Negative screening tends to be used to identify essential genes and genes required for transformation by oncogenic viruses such as Epstein Barr virus (EBV) or in cancer, whereas positive screens are used to identify resistance to toxins or pathogen infections [13].

Of the many genome-wide pooled sgRNA knockout libraries available for the human genome, two have been predominantly adopted; the activity-optimised genome-wide library by Sabatini and Lander which has 10 sgRNAs per gene and 187535 sgRNAs in total [44] and GeCKOv2 by Zhang *et al*. which has 6 sgRNAs per gene and 123411 sgRNAs in total [45]. Two second generation libraries have subsequently been designed to optimise the specificity of the sgRNAs. These are the Toronto KnockOut library by Moffat which has 12 sgRNAs/ gene and 176500 sgRNAs in total [46] and the Brunello genome-wide library by Doench *et al*. which has 76441 sgRNAs in total with four sgRNAs/ gene [47]. The ability to design custom sgRNA libraries for a selected gene set is also available. This allows focused screens within an area of interest; for example, the antiviral response, which will be further discussed.

**Fig. 2.**
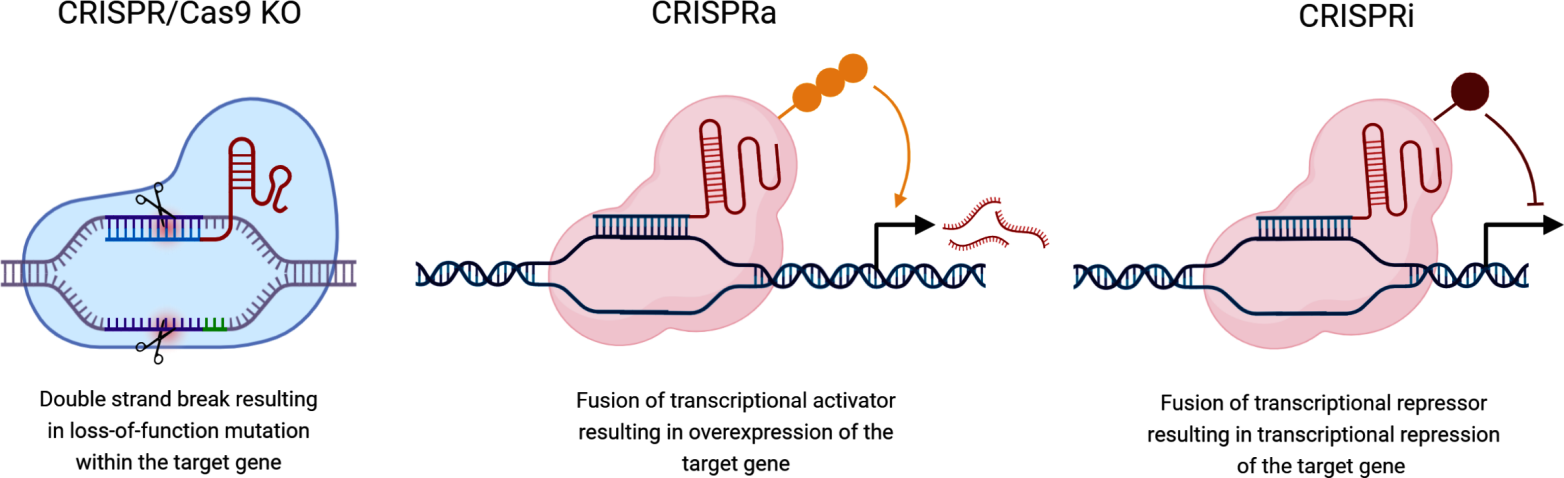
Different approaches to CRISPR/Cas9 screening based on Cas9 variation. In CRISPR/Cas9 KO screens, a single guide RNA targets the Cas9 endonuclease protein to a specific locus in the coding sequence of a gene leading to a double strand break in the DNA. This activates the cellular DNA damage response; primarily, the imprecise non-homologous end-joining pathway that introduces INDELs resulting in gene knockout. For CRISPR activation (CRISPRa) and CRISPR interference (CRISPRi) screens, fusion of either a transcriptional activator or repressor domain to a catalytically inactive Cas9 subunit results in overexpression or inhibition of gene expression, respectively. Created with biorender.com.

Alongside the CRISPR/Cas9 system for creating knockouts, a catalytically dead version of Cas9 (dCas9) has been developed which can be used for CRISPR activation (CRISPRa) and CRISPR interference (CRISPRi) (Fig. 2). dCas9 is programmed with a sgRNA but rather than mutating genomic loci, it occupies genomic sites (reviewed by Wang *et al.* [48]). In CRISPRi, dCas9 is covalently bound to a repressor domain, such as KRAB, resulting in transcriptional repression of the target gene by the promotion of heterochromatin at the promoter [49, 50]. Conversely, CRISPRa results in transcriptional activation for overexpression of genes. This can be achieved through multiple approaches including the fusion of VP64, p65 and RTA [51]. CRISPRi is similar to RNAi in that it causes knockdown however currently, no CRISPRi screens have been used to investigate virus–host interactions. Likewise, CRISPRa may be likened to the overexpression screening as it results in an upregulation of gene expression whilst enabling overexpression of large genes to occur in a pooled format.

### Using pooled CRISPR/Cas9 screening to identify antiviral factors

CRISPR/Cas9 screening to study virus–host interactions has primarily been performed in a pooled format. For example, Richardson *et al*. carried out a pooled screen to investigate ISGs implicated in the innate immune response to flaviviruses [52]. Using the Brunello genome-wide knockout library, interferon alpha-inducible protein 6 (IFI6) was identified as an ISG responsible for inhibiting yellow fever virus (YFV) upon pre-treatment with IFN. MAGeCK was used to identify three significantly enriched sgRNAs, *IFI6*, signal transducer and activator of transcription 2 (*STAT2*) and interferon regulatory factor 9 (*IRF9*), these results were validated through individual knockouts. IFI6 was also ectopically expressed, with the results demonstrating that the absence of IFI6 permits viral infection and its presence significantly restricts it. Hits were further shown to be antiviral against additional flaviviruses; West Nile virus (WNV), dengue virus (DENV) and zika virus (ZIKV) [52]. This is unlike many pooled virus–host interaction screens that use survival screens to identify proviral factors.

CRISPR/Cas9 screening has been applied to identify host factors involved in severe acute respiratory syndrome coronavirus 2 (SARS-CoV-2) pathogenesis, the virus responsible for the global COVID-19 pandemic that emerged in 2019. Given the nature of global pandemics, the need to rapidly identify host-dependency and antiviral factors that have the potential to be exploited is crucial. At the time of writing, at least three independent screens have been performed and two of which have tested small molecule inhibitors against candidate genes. Wei *et al*. performed a genome-wide screen in Vero-E6 cells using an African Green Monkey library, used due to their susceptibility to SARS-CoV-2 infection, to successfully identify genes resulting in both resistance and sensitisation to virus-induced cell death [53]. Three of the most highly ranked antiviral genes identified by the screen are components of the histone H3.3 chaperone complex. An independent genome-wide screen using the GeCKOv2 library was performed by Daniloski *et al*. in A549 cells constitutively expressing the angiotensin-converting enzyme 2 (ACE2) receptor [54]. Several sets of related genes were identified including four members of the endosomal protein sorting Retromer complex and four members of the endosomal trafficking Commander complex. Further gene set enrichment analysis established endosomal regulation and function as a consistent theme within candidate genes. Both studies subsequently tested small molecule inhibitors against a selection of candidate genes. Wei *et al*. identified three molecules that inhibit SARS-CoV-2 replication and virus-induced cell death and Daniloski *et al*. identified seven inhibitors that resulted in more than 100-fold reduction of viral load with combinatorial therapy with small molecule inhibitors showing an additive effect in protection against SARS-CoV-2 infection. A third screen by Hoffmann *et al*. [55] used a focused screen established from previously reported SARS-CoV-2 protein interactors [56]. Using this focused screen, they identified host factors that promote and antagonise SARS-CoV-2 and three related coronaviruses at 33 and 37 °C, the biological temperatures of the upper and lower respiratory tracts respectively. A number of genes implicated in promoting SARS-CoV-2 infection, including Rab GTPases, and antagonising virus induced cell death, including mammalian mitochondrial ribosomal small subunit (MRPS) genes, were identified. The use of both genome wide and focused screening has therefore identified sets of genes, alongside individual genes, implicated in affecting the pathogenesis of SARS-CoV-2, something that may have otherwise not been identified in single-gene studies. In a situation requiring fast and actionable results, particularly in identifying therapeutic targets, high-throughput screening has provided a means to do so through providing targets for the repurposing of current pharmacological molecules.

### CRISPR/Cas9 loss-of-function screening provides a synergistic view of HIV-1 infection

Many screens, and previous reviews, have focused on virus–host interactions upon infection with flaviviruses [18, 52, 57–60]. However, a multitude of other viruses have also been investigated including human immunodeficiency virus type 1 (HIV-1). Screens to investigate the interplay between host genes and viruses have identified various aspects of infection, including host dependency, antiviral and latency promoting factors.

Different cell lines have been utilised to study various aspects of HIV-1 infection. Park *et al*. performed the first genome-wide CRISPR/Cas9 screen in a CD4^+^ T cell line model to identify HIV-1 host dependency factors due to its susceptibility to HIV-1 infection [61] whilst Rathore *et al*. used the J-Lat 10.6 cell line to identify novel latency promoting factors in HIV-1 infection [62]; a model Jurkat T-cell-derived cell line used as a model for HIV-1 latency, containing a single integrated, replication-incompetent HIV-1. Both screens used a green fluorescent protein (GFP) reporter for virus infection. Host dependency factors were identified by Park *et al.* by isolating GFP-negative cells upon transduction of cells with a custom sgRNA library, subsequently identifying five significantly enriched genes; *CD4*, *CCR5*, *TPST2*, *SLC35B2*, and *ALCAM*. In the virus latency study conducted by Rathore *et. al*, GFP expression correlated to HIV-1 reactivation and replication. Alternatively, GFP-positive cells were sorted by FACS for sgRNA enrichment analysis by MAGeCK resulting in fifty-two latency-promoting genes being selected for further validation. Host dependency hits were validated by single cell knockout with the loss of either TPST2 or SLC35B2 conferring lack of viral entry which was restored upon the reintroduction of each gene. Latency promoting factors were validated using small molecule inhibitors, characterising the role of 26S proteasome non-ATPase regulatory subunit 1 (PSMD1) in HIV-1 latency through reactivation of HIV-1 infection following treatment.

To investigate antiviral factors targeting HIV-1, Ohainle *et al.* developed a human ISG CRISPR/Cas9 knockout library targeting approximately 2000 antiviral genes with sgRNAs sourced primarily from existing libraries [63]. It was first used alongside a custom vector in a CRISPR/Cas9 knockout screen to identify HIV-1 restriction factors. Ohainle *et. al* modified the lentiCRISPRv2 vector to maintain a complete HIV-1 LTR (HIV-CRISPR), maintaining the transcriptional and packaging competency of the vector whilst also delivering Cas9 and the sgRNA. Consequently, sgRNAs targeting antiviral genes were enriched in the viral supernatant due to increased viral replication in knockout cells. The screen was performed in zinc-finger antiviral protein (*ZAP*) knockout cells to increase screen performance as it had been suggested that ZAP inhibited packaging of the HIV-1-CRISPR vector in viral particles, due to the high GC dinucleotide content of the HIV-1-CRISPR genome, therefore reducing the release of HIV-1-CRISPR virions. Genes including IFN-α receptor subunit 1 (*IFNAR1*), *STAT1, IRF9*, *MxB*, *IFITM1*, *Tetherin* and *TRIM5α* were identified but it was suggested that rather than a single ISG having a dominant effect, the cumulative effect of multiple ISGs contributes to the restriction of HIV-1 replication. Ohainle *et al*. subsequently repeated the screen with different isolates of HIV-1 and demonstrated strain dependency of ISGs [63]. Results of the investigation looking at different HIV-1 isolates are consistent with those of Schoggins *et al*. and the generally accepted view of how IFN-dependent antiviral restriction operates [23]. Statistical analysis of enriched sgRNAs identified ISGs with modest antiviral phenotypes enabling recapitulation of patterns observed with overexpression screening. This shows the power of genome-wide CRISPR/Cas9 knockout screening in answering fundamental questions about the antiviral response.

As has been shown, many factors are able to help guide the design of a CRISPR/Cas9 screen and direct the outcome of results. Factors including library choice, cell line and vector can be manipulated to study different aspects of virus–host interactions highlighting the diversity and flexibility of the technique. Various aspects of the interplay between virus and host have been identified using varying experimental protocols which together provide a more rounded picture of the virus-host interactions during infection.

## Overexpression screening

Overexpression screening allows for the characterisation of genes through observing how enhanced expression of a gene affects phenotype. The development of an ISG lentivirus library, consisting of 389 different ISGs, by Schoggins *et al.* in 2011 enabled the identification of antiviral factors, and their effects on viral replication, through overexpression of these ISGs in cells infected with a virus [23]. In an arrayed format, cells are transduced with a lentiviral vector, resulting in ISG-TagRFP expressing cells before being infected with GFP-expressing virus. Quantification of viral replication is performed using FACS by gating the TagRFP-positive population and determining the number of green fluorescence protein positive (GFP+) cells in this sub-population [23].

The current ISG overexpression screening platform is not able to distinguish regulatory from direct antiviral ISGs. In order to overcome this and distinguish between ISGs that have a regulatory function and those that act directly on a virus, Kane *et al*. used an interferon-stimulated response element (ISRE) driven reporter cell line. This enabled directly acting ISGs to be identified as they would not activate the reporter, unlike transcriptional regulators such as IFN regulatory factor 1 (*IRF1*) and toll-like receptor 3 *TLR3*. This reporter activity was measured soon after transduction to minimise indirect effects [64]. It is also vital in overexpression screening to ensure that the phenotypic effect is a result of the overexpressed ISG and not additional ISGs induced as a result of transduction and/or infection. This is resolved with the use of STAT1-deficient cells and upon investigation of varying cell types, Schoggins *et al*. showed that STAT1-deficient fibroblasts exhibited the highest levels of transduction compared with other cell types [23]. Similarly, a recent publication described using an arrayed ISG overexpression library to identify antiviral factors that inhibit human cytomegalovirus (HCMV) by performing parallel screens in human primary fibroblast cells and IRF3-deficient fibroblast cells [65]. IRF3-deficient cells provided confidence that the ISGs identified were specific against HCMV and not produced as a result of non-specific activation of IFN signalling. This further enabled identification of previously unknown IRF3-dependent ISGs. Using this method they were able to identify TRIM25 and ZAP as HCMV inhibitors amongst other proteins [65].

### Identifying restriction factor patterns with comparative overexpression screening

Multiple studies have used ISG overexpression screening in a comparative manner to investigate differences in antiviral effectors between viruses, either between virus families or within the same family. Schoggins *et al*. investigated ISG inhibition patterns across viruses from three families and identified broad-acting inhibitors, such as *IRF1*, alongside specific inhibitors. In the same study a predominant additive antiviral effect of ISGs was identified through varying combinatorial pairing of hits from the primary ISG screen [23].

In 2014, further comparative ISG analyses were carried out on 13 diverse viruses comprising of six negative-sense single strand RNA (−ssRNA) viruses and seven positive-sense single strand RNA (+ssRNA) viruses [66]. Hierarchical clustering analysis was performed using R statistical software for viruses screened in STAT1^−/−^ fibroblasts, thus ruling out the potential complication of endogenously expressed ISGs as a result of transduction and/or infection. Infectivity of the 13 viruses was assessed in cells overexpressing 22 ISGs and the subsequent hierarchical clustering analysis grouped viruses and ISGs to graphically show either hierarchical relationships or similar ISG effects on virus infectivity via dendrograms or heatmaps respectively. Results revealed two clusters corresponding to −ssRNA and +ssRNA viruses, indicating that related viruses are targeted by similar ISGs. Sub-clusters formed between more phylogenetically similar viruses; for example, within the −ssRNA cluster, a sub-cluster containing respiratory syncytial virus (RSV), human metapneumovirus (HPMV), parainfluenza virus subtype-3 (PIV3) and measles virus (MeV) is formed. It is unsurprising that similar ISGs are antiviral against these viruses given that RSV and HPMV are both Pneumoviruses whilst PIV3 and MeV are both Paramyxoviruses, two families that were only taxonomically separated in 2016 [67]. Similar to the −ssRNA viruses, predicted clustering is seen in the +ssRNA viruses with YFV and WNV, both flaviviruses, showing strong similarity. Additional hierarchical clustering identified the same pattern of negative and positive-sense clusters; the top 30 hits identified in the primary screen were stated as present or absent for the selected 12 viruses and the binary values were subsequently analysed using the MATLAB Statistics Toolbox. This observation was only possible due to the adaptability of overexpression screening to be used to investigate different viruses.

### Overexpression screening using human and macaque ISG libraries

In 2016, the original overexpression library, consisting of 389 different ISGs, was expanded to include a library of ISGs from Rhesus macaques [64]. Combined, this library constitutes 488 different ISGs, with 252 genes having both human and macaque variants. For comparison, the CRISPR/Cas9 ISG library contains approximately 1900 genes [63]. This combined human–macaque library was further expanded in 2019 by Rihn *et. al* to exceed 500 and 300 ISGs from humans and macaques, respectively [68]. Many overexpression screens have taken the approach to use the combined human and macaque ISG library despite investigating human pathogens.

Feng *et al*. used a flow cytometry gain-of-function screen to investigate ISGs involved in restricting early stages of the life cycle of the Bunyamwera orthobunyavirus (BUNV), the prototype virus of the larger Bunyaviridae order [69]. Cells overexpressing a combined human and macaque library [64] were infected with GFP-expressing BUNV (BUNV-EGFP). Subsequently, 20 inhibitory ISGs were identified, with the expression of nine human and five macaque genes reducing BUNV-EGFP titre by more than five-fold compared with the empty vector control. Of the 20 hits, 13 genes had isoforms in both libraries, thereby validating the use of macaque genes in investigating the human immune response. The inhibitory capabilities of these genes, both human and macaque orthologues, were tested against 15 different bunyaviruses from four different families, including both clinical and agricultural pathogens. Despite divergent effects of the ISGs between bunyaviruses, fold inhibition of viruses by the human and macaque orthologues remained similar. IFN stimulated gene 20 (*ISG20),* a well-documented antiviral protein against many different viruses [70], was one of the genes validated. Both the human and macaque orthologues of *ISG20* significantly inhibited at least 9 of the 15 viruses by varying amounts. The observed varying activity between the human and macaque *ISG20* orthologues, also seen in the BUNV fold inhibition rankings of the other 19 hits, may indicate that non-human isoforms have divergent anti-bunyaviral specificities. This species-dependent variation in ISGs had previously been identified by Kane *et. al* when testing the antiviral activity of ISGs against 11 different retroviruses with more than one third of hits identified present only in the macaque library [64].

Without screening, and the availability of both a human and macaque library, orthologues with similar antiviral capabilities would not have been identified. This is important as the use of dual screening libraries may not only identify additional antiviral proteins but provide potential insights into the differences in the IFN response between species that may aid or protect against zoonotic transmission of viruses. Sequence differences in the macaque render some virus countermeasures ineffective. This approach may therefore be able to identify ISGs that are countered by the virus.

## RNAi screening

RNA interference (RNAi) was first discovered in *Caenorhabditis elegans* in 1998 [71] and was first used for genome-wide screening in 2008 to investigate host dependency factors for influenza virus replication [72]. Since then, it has been used to investigate fields including cancer biology and signalling alongside infectious disease, encompassing virus–host interactions. Such studies have been carried out *in vitro* in mammalian cell culture systems and *in vivo* in model organisms, including mice and *Drosophila melanogaster* (reviewed elsewhere [73, 74]). The performance of RNAi screening across multiple organisms is enabled due to the conserved nature of the RNAi system across most eukaryotes. In brief, RNA constructs termed small interfering RNA (siRNA) or short hairpin RNA (shRNA) are introduced into cells, resulting in the silencing of target genes by degrading mRNA transcripts in the cytoplasm. shRNA, following processing by Drosha, is exported to the cytoplasm to be processed by Dicer, an endoribonuclease, to produce siRNA. This is then loaded into the RNA-induced silencing complex (RISC) and the siRNA–RISC complex can cleave complementary mRNA. siRNA is synthetically synthesised and can be directly transfected into the cytoplasm, therefore bypassing processing steps, to be directly loaded into the RISC (reviewed [74, 75]).

### Inconsistencies within RNAi screening

Many arrayed siRNA screens have been performed to identify host factors (HFs) that contribute to viral infection and multiple RNAi screens investigating the same virus have highlighted a lack of overlap in hits identified. Aydin *et. al* identified HFs required for human papilloma virus type 16 (HPV16) infection using pseudoviruses, which allowed them to focus on viral entry, but of the 162 HF hits identified, only four overlapped with the 261 hits previously identified by Lipovsky *et.al* to contribute to HPV16 infection [76, 77]. Similarly, Beard *et. al* identified factors that influenced vaccinia virus (VACV) replication, both proviral and antiviral, and when hits were compared with two previous VACV RNAi screens, no hits were identified in all three studies [78–80]. A study investigating host factors involved in Paramyxoviridae and Pneumoviridae life cycles showed discrepancies in identified hits dependent on the analysis tools used [81]. Independent screens were performed on MeV, mumps virus (MuV) and human respiratory syncytial virus (hRSV) and the data was subsequently analysed via different methods; meta-analysis approaches through ranking of z-scores and a bioinformatics approach using the Kolmogorov–Smirnov test. Of the 42 proviral genes that overlapped between all three viruses as identified by complementary meta-analysis, only 24 of these were also identified by the bioinformatics package.

Additionally, multiple screens have been performed to identify host factors required for HCV infection. Studies have investigated different stages of the viral lifecycle through use of different experimental systems. Tai *et al*. [82] and Ng *et al*. [83] used a replicon system enabling identification of host factors implicated in virus replication whilst Li *et al*. [84] used an infection competent HCV cell culture system (HCVcc) enabling investigation of all stages of the viral lifecycle. Little overlap in hits was seen between the two screens and the differences in study design may have contributed to this. The replicon systems used in these studies were not analogous as Tai *et al*. used a replicon encoding viral proteins NS3 to NS5B and a firefly luciferase reporter [82] whilst Ng *et al*. used a replicon system with a secreted alkaline phosphatase (SEAP) reporter [83]. Both replicon screens identified *SLC12A5* as a hit, however, Tai *et al*. used a liver cell line and this gene is a neuron-specific transcript leading to the assumption the gene was a false positive caused by off-target effects in more than one screen [82]. Another genome-wide screen was performed by Fusco *et. al* to identify IFN effector genes that inhibit HCV [85]. Of the candidate genes identified, only 52 % of the hits had previously been shown to have antiviral activity, with only 8 % previously having been described as having antiviral activity against HCV. The use of pseudovirions expressing HCV glycoproteins identified four antiviral genes (*DPP4*, *MYST1*, *PPP3CB* and *SLC7A2*). Four genes (*ALG10*, *DPP4*, *PPP3CB* and *PDIP1*) were also found to restrict viral replication by using HCV full genome OR6 replicons.

Results from previous studies have indicated that the lack of overlap in candidate genes between screens of the same virus is due to differences in screening methodology [82]. However, results from additional studies indicate that even in the absence of screening differences, the choice of analysis can be enough to influence identified hits, therefore contributing to inconsistencies in results – a phenomenon that may also be present in CRISPR/Cas9 screening analysis. Alternatively, despite the lack of overlap in individual genes between studies, bioinformatics analysis is able to identify conserved pathways between hits in different screens [76]. For example, nuclear pore proteins have been identified as HFs in multiple RNAi screens investigating VACV, indicating their role in Poxvirus infection [78]. This may indicate a role for comparative RNAi screening in understanding the broader picture of virus–host interactions.

### RNAi screening identifies antiviral factors restricting flavivirus replication

Alongside the popularity of CRISPR/Cas9 screens for investigating flavivirus restriction factors, RNAi screening has also been performed to identify restriction factors against this family of viruses. Following a genome-wide siRNA screen to identify antiviral factors restricting influenza A virus (IAV) infection, IFITM proteins were identified as hits [86]. These were subsequently tested against several flaviviruses; WNV, YFV and Omsk haemorrhagic fever virus (OHFV). Using virus-like particles expressing the envelope proteins of each respective virus, IFITM1, IFITM2 and IFITM3 were all able to inhibit infection compared with controls. IFITM3 was validated as a flavivirus antiviral factor upon infection with pathogenic WNV and DENV-2. Stable overexpression and siRNA knockdown of IFITM3 resulted in significantly decreased and increased viral replication, respectively. More recently, Li *et al*. used an ISG specific library to investigate WNV restriction factors [87]. Adapted from microarray studies, the ISG library contains shRNA against 245 genes. The shRNA is cloned into a lentiviral vector containing a GFP reporter. Following successful transduction, the cells are then challenged with a red fluorescent protein (RFP)-expressing virus similar to the overexpression screening method [87]. Using the ISG-specific library, thirty ISGs were identified whose knockdown significantly increased infectivity. These hits were validated through ectopic gene expression. Genes such as IFI6 were shown to restrict viral replication, an expected result as IFI6 has been shown to be widely antiviral. IFI6 has subsequently been shown to be a flavivirus restriction factor by Richardson *et al*. using a CRISPR/Cas9 screen [52]. IFITM3, an antiviral factor previously identified by Brass *et al*. to restrict WNV and DENV-2 infection [86] was not identified as a hit using the ISG library. However, Li *et al*. subsequently showed that IFITM3 was able to to restrict WNV when ectopically expressed. This reaffirms the need for independent validation of hits due to inconsistency between screen designs and resulting antiviral factors identified. An interesting finding by Li *et al*. was the identification of a regulator of cellular gene expression; activating signal cointegrator 1 complex subunit 3 (ASCC33) [87]. Overexpression of the gene increased WNV replication whilst silencing decreased replication through the upregulation of multiple antiviral ISGs, the inverse effect seen with other ISGs. It was suggested that following IFN-β induced expression of ASCC33, ASCC33 modulates the activity of IRF-3 and IRF-7 pathways to dampen ISG expression. Among ISGs there are proviral and antiviral factors, however, during screening using a genome-wide library, genes permitting virus replication are often classed as host dependency factors whilst genes restricting virus replication are termed antiviral factors. Overexpression of ASCC33 results in a host dependency factor-like phenotype, however, by narrowing the library to ISGs only, it was instead revealed to be a modulator of the IFN response.

## Choosing a screening method

### Advantages and disadvantages of screening techniques

Advances in molecular tools, such as the development of NGS for sequence-based analysis, have enabled forward genetic screening to become easier. These advances are therefore imperative as the success of forward genetics relies on the ability to characterise genes resulting in the desired phenotype. For the three screening methods discussed, there are advantages and disadvantages to each (summarised in Table 1) and many other pitfalls are due to mechanistic details of the methods previously mentioned (reviewed elsewhere [23, 37, 74, 75, 88]). The decision of what screen to use for an investigation may therefore be influenced by what the screen is able to achieve.

**Table 1.** Summary table of advantages and disadvantages for each of the three screening methods discussed; CRISPR, RNAi and overexpression screening

	Advantages	Disadvantages
**CRISPR/Cas9**	Permanent targeting of genes for robust response. Ability to multiplex. Ease of construction. Adaptability of 20 bp protospacer. Target specificity. Eliminates confounding effects from low level protein expression. Use of dCas9. Reduces activation of innate immune response compared with RNAi. Well established in mammalian cell culture.	Cannot be used to study essential genes. Relies on Cas9 expression levels. Requires selection step. Difficult to identify moderately acting antiviral factors. Off-target effects. Redundancy.
**RNAi**	Ability to investigate essential genes. Ability to restore protein expression for validation. Reagents readily available. Well established in mammalian cell culture, mice, and Drosophila models.	Off-target effects and high rate of false-positives. Low percentage of reproducible hits. Inability to knockdown non-coding regions. Incompleteness of gene knockdown. Longer siRNAs can trigger the immune response. Redundancy.
**Overexpression**	Combined species libraries (human and macaque). Suitability for arrayed comparative screening. Library limited to ISGs. Able to identify moderately acting antiviral factors.	Dependent on producing high lentiviral stocks. ISG-mediated and overexpression artefact toxicity. Requirement for fluorescence readout. Requirement for automation of equipment. Cannot identify proteins that function in a complex. ‘Long genes’ more difficult to handle.

A large influencing factor for screening is the range of the available libraries. The library of the overexpression screening described currently is limited to ISGs [2], and is therefore limited to investigating antiviral factors whilst CRISPR/Cas9 and RNAi screening are also able to identify novel host dependency factors. Conversely, the overexpression library has the benefit of possessing a combined library of human and macaque genes for comparative studies [64]. Likewise, although this review focusses on RNAi screening in mammalian cell culture systems, there are well established libraries and protocols for RNAi screening in *Drosophila* [89]; an advantage over other screening.

A widely accepted caveat with RNAi based screening is the presence of off-target effects (OTEs) [90] which fall into three categories; OTEs caused by siRNA sequence similarity [91, 92], OTEs caused by microRNA (miRNA) like events [93, 94] and sequence independent OTEs [95]. The resolution of this problem is important in screening as both false negative and false positive results lead to reduced reproducibility between RNAi screens [96]. OTEs caused by siRNA sequence similarity occur when a siRNA sequence is too similar to an unrelated messenger RNA (mRNA). Whilst most siRNAs are 21–23 nt [74], a 19-mer rule has been established to counter this. It has been shown that a 19 nt siRNA is sufficient to result in gene knockdown. However, to reduce the likelihood of OTEs, the 19-mer must not show any sequence similarity with any other mature gene transcript [74, 97]. Additionally, most siRNA libraries contain at minimum three siRNAs per gene and in most studies a hit is only considered when two or more of these give rise to a result that is two to three standard deviations away from the sample set mean [98, 99].

OTEs caused by miRNA-like events occur due to the shared downstream effector, Ago, of siRNAs and miRNAs. Whilst complete complementarity of an siRNA to target sequence results in on-target cleavage, partial pairing due to high complementarity of the seed sequence with the target sequence [100], which is observed in miRNA–target interactions in animals, results in unintended miRNA-like OTEs with the use of siRNAs [101]. Unfortunately, unlike siRNA similarity, this is a harder obstacle to overcome when designing siRNAs for screening. The last category of OTEs are sequence-independent events. It has been found that siRNAs are able to activate the type I IFN response in a sequence-independent manner [95]. RNAi screens can also be performed using dsRNA however this is not used in mammalian cell screens as dsRNA is a PAMP that is recognised and subsequently activates IFN induction [102]. CRISPR/Cas9 screening displays a reduced immune response compared with RNAi screening due to a reduced susceptibility to OTEs.

Multiple developments have been made in the design of sgRNAs for CRISPR/Cas9 screening to reduce the likelihood of OTEs and increase target specificity [45, 47, 103, 104]. In comparison, overexpression screening does not require constructs for the knockdown or knockout of genes. This therefore removes the obstacle of hits produced as a result of OTEs in the screen. An important distinction to be made between RNAi and CRISPR/Cas9 screening is the duration of genetic modulation and the point in the cell at which each technology targets. Genetic modification of genes by Cas9, rather than dCas9, results in the permanent targeting of genes at the DNA level [35] whilst RNAi results in a transient reduction in gene levels at the mRNA level [105]. This means protein expression can be restored in the cell, allowing for validation of hits identified by RNAi screening [106]. However, a limitation to this transient knockdown of mRNA is the incompleteness of gene knockdown. In a comparison of a CRISPR GeCKO screen, with six sgRNAs per gene, and a shRNA screen, with four shRNAs targeting each gene, the shRNA screen showed incomplete knockdown of fluorescence whilst the GeCKO screen resulted in 93 % of cells showing no fluorescence [107]. A further consequence of acting at the mRNA level is also the inability to knockdown non-coding regions, unlike CRISPR/Cas9 screening [108]. There is also a desire to perform these screens in primary mammalian cells due to their increased physiological relevance. However, due to the limited time-span these cells can be cultured for, there are difficulties surrounding screening which, from editing to analysis, can often be lengthy. Transduction of primary cells is often difficult with low resulting transduction rates meaning perturbational studies, such as RNAi, and CRISPR/Cas9, involving lentiviral transduction, are more difficult than in cultured cell lines [109, 110]. Additionally, cell viability or growth screening, as is often the case with CRISPR/Cas9 screens, is difficult. Alternative cellular markers or FACS-based phenotypic selection, as shown with primary T-cells, may be used instead. There are advantages to using both immortal and primary cell lines during screening. Immortal cell lines have a less limited passage number, allowing for a growth-based phenotypic readout, whilst primary cells can be used to identify critical signalling pathways or effector functions. For example, morphological or functional differences in human immune cells may result in unidentified pathways if other cell lines are used. It has been suggested that for some genes, knockdown of a gene by shRNAs or siRNAs may result in an alternative or reduced phenotype compared with complete knockout [111, 112]. Therefore, if performing a positive selection screen and sorting cells with FACS, of which only the extreme phenotypes are selected for, knockdown may cause significant results to be missed [113]. A knock-on effect of this is the strength of antiviral factors each screen can identify. Knockdown, as a result of RNAi, is incomplete and so may not be able to identify moderately acting antiviral factors whereas overexpression and CRISPR/Cas9 screening, which results in complete knockout, have a greater capacity to identify moderate antiviral factors or those that work in combination with others [63].

Limitations of overexpression screening remain the difficulty in identifying proteins that function in a complex and the cost of generating libraries. The overexpression of a single subunit may not evoke a phenotypic result and so factors may be missed; this is an issue knockout screens are able to overcome. Equally, CRISPR/Cas9 and RNAi screening may be insufficient when trying to investigate redundant factors or when investigating antiviral factors that target a virus not susceptible to IFN due to the reliance on extreme phenotypes for the identification of hits. If more than one gene product restricts infection, ‘deleting’ only a single gene will be phenotypically silent or the effect will be so minimal that the cell is not selected for further processing.

If a virus has reduced susceptibility to IFN, it is more difficult to discern functionally redundant ISGs from ISGs with a moderate effect due to the vast number of ISGs expressed. This is because the difference in phenotype compared with the control, or ‘window of identification’, is reduced. As overexpression screening does not rely on the restoration of infection, it is better equipped to identify ISGs of moderate effect. By broadening the window of identification, it would be possible to increase the number of moderately acting antiviral factors identified in CRISPR/Cas9 screening. One possibility might be to use cells deficient in IFN stimulated gene 15 (ISG15); these cells, when treated with IFN-α, exhibit an overamplified IFN response resulting in the overexpression of ISGs and, subsequently, a greater ability to resist virus infection. Holthaus *et al*. confirmed that virus resistance in ISG15^−/−^ cells was due to the overexpression of viral restriction factors [114]. Because cells are resistant to infection, they are phenotypically silent and not detectable by FACS when infected with a reporter virus; deletion of a restriction factor, even one with low to moderate activity, results in a detectable signal that would be otherwise missed. For example, previously published work has indicated that IFIT1 modestly inhibits human parainfluenza virus type 2 (hPIV2) protein expression, at least in cell-free assays [115]. Despite this, in an infection model (as would be used in a screening protocol), IFIT1-deficient cells pre-treated with IFN showed levels of viral protein expression not overtly different from IFN-pre-treated control cells. However, when IFIT1 was knocked down in ISG15^−/−^ cells, hPIV2 protein expression was significantly higher compared with IFN-treated ISG15^−/−^ cells with intact IFIT1 expression, indicating that IFIT1 does indeed play an antiviral role, albeit with low to moderate activity, against hPIV2 [114]. Such conclusions could not be made without expanding the ‘window of identification’. Nonetheless, positive selection CRISPR/Cas9 screens cannot be used to study essential genes, as knockout of the genes is lethal to the cell, therefore removing them from the set of genes subsequently analysed [13, 42, 43].

Both RNAi and CRISPR/Cas9 screening libraries and reagents are readily available, however, the adaptability and ease of construction of the 20 bp protospacer enable a greater flexibility in designing custom CRISPR/Cas9 libraries [116, 117]. Despite this, both overexpression, CRISPR/Cas9 and RNAi screening, if using shRNA, primarily rely on the production of lentiviral stocks and efficient transduction into cells. CRISPR/Cas9 screening further relies on Cas9 expression levels in the cell if a single transduction vector is not used [118].

Despite the advances in gene perturbation screening since its development, there are still pitfalls associated with the bioinformatic analyses used to dissect such screens, not only limited to the technical noise generated by such a high-throughput technique. The popularity of CRISPR/Cas9 screening is increasing at a faster rate than the development of data analysis methods [119]. Currently there is no consensus bioinformatic analysis method of such screens with many algorithms being more suited to, or originally designed for, siRNA screens, RNA-seq and microarrays rather than CRISPR/Cas9 [120]. Despite this, MAGeCK, alongside other algorithms, have been developed specifically for analysis of CRISPR/Cas9 screens [121]. The large range of bioinformatic tools currently available results in varying modelling strategies, with different strengths and weaknesses, being utilised between analyses which users may be unaware of [119]. Some analysis packages, such as CasTLE and BAGEL, require the input of gold standard genes (927 non-essential genes derived from previous pooled screens) whilst others, such as MAGeCK, do not [122]. The required negative control has also been suggested to affect the presence of false positive hits in the bioinformatic pipeline; some strategies and computational methods use non-targeting guides as negative controls in place of sgRNAs targeting non-essential genes. It has been suggested that the latter may result in fewer false positives [123]. Additionally, most algorithms designed to analyse CRISPR genes assume that most genes are non-essential. Whilst this does not appear to affect the results of large genome-wide screens it is currently not known what effect this has on the results of custom screens whose targeted genes are under selection [123]. As previously discussed, differences in the bioinformatic analysis of RNAi screens contributes to observed variance in hits [81] and the same phenomenon may be occurring during the bioinformatic analysis of CRISPR/Cas9 screens.

### Using complementary approaches within screening studies for hit identification

As previously described, each of the screening methods discussed have their advantages and disadvantages. There are situations when a single technique cannot be used as results would be missed. There is therefore scope to use these techniques in a complementary fashion, to result in a more comprehensive picture of virus–host interactions.

Simultaneous RNAi and CRISPR/Cas9 screening has been used to investigate the effects of an antiviral drug and showed differences in significant hits [124]. Essential genes, such as those in the nucleotide biosynthesis pathway, were significant hits in the siRNA screen whilst a CRISPR/Cas9 screen identified others, such as those in mTOR signalling and regulation that are non-essential but require complete deletion. Kranz *et al*., although not investigating virus–host interactions, used a complementary approach to screening using both RNAi and CRISPR/Cas9 technology. Results of the primary screen identified FAT Atypical Cadherin 1 (FAT1) as having a role in apoptosis. This was independently tested using CRISPR/Cas9 knockout and both approaches resulted in the same phenotype, validating the result [125]. Subramanian *et al*. used a complementary approach to investigate the antiviral action of Tudor domain-containing protein 7 (TDRD7) against Sendai virus (SeV). TDRD7 was identified as an antiviral factor following a shRNA screen using a library against human ISGs. Following knockdown of the gene by RNAi, validation of the hit and further characterisation of the resulting phenotype was performed by knocking out the endogenous gene using CRISPR/Cas9 and ectopically expressing an exogenous version of the gene [126].

In each of these examples, the use of two gene perturbation technologies within the same study allowed for independent validation of results and further characterisation of hits from primary screens. In these examples, CRISPR/Cas9 was used to validate RNAi hits, however, the reverse could be performed. The use of RNAi following CRISPR/Cas9 screening may allow for the strength of hits to be validated when comparing phenotypes resulting from knockout and knockdown of the same gene. The use of RNAi in validating CRISPR/Cas9 screens may also determine if the resolution of a gene, as RNAi is temporary, reverses the observed phenotype back to wildtype.

## Other approaches

Techniques other than those discussed have also been used to study virus–host interactions. These include, data and literature mining, single-cell approaches and proteomics approaches. McDonald *et al*. curated a single combined database of genes from published studies using omics approaches to investigate genes upregulated in response to RSV infection. From this database they identified genes present across studies and ranked them by factors including occurrence to generate a candidate list of genes to be validated [127]. The use of a literature-mining approach to generate a candidate list enables identification of gene overlap, a pitfall of some screening methods, to provide confidence in hits identified. Single-cell approaches have also been used to investigate host–pathogen interactions. Single-cell RNA-seq (scRNA-seq) has been used to identify genes associated with latent HIV-1 infection [128, 129]. Results indicated an increase in HIV-1 suppressive genes, such as chemokine Ligand 3 (*CCL3*), and a decrease in IFN responsive genes, including *STAT1*, *IFI6* and ISG20. However, single-cell approaches are often limited to arrayed formats, resulting in reduced throughput. CRISP-seq, also referred to as CROP-seq and Perturb-seq, has been developed to encompass the resolution of RNA-seq and the high-throughput nature of pooled CRISPR/Cas9 screening [130–133]. This allows for a more detailed understanding of cellular interactions caused by perturbations outside of phenotypes such as cell survival. CRISP-seq has been used to investigate the effect of knocking out IRF9, a gene within the antiviral IFN response [133]. It was found that genes enriched for IRF9 knockout had a reduced antiviral response, including the genes *IFIT2*, *CXCL10* and *OASL1*. This shows the power of using CRISP-seq to identify genes involved in regulating virus–host interactions. cDNA screening, although not predominantly used nowadays, is another gain-of-function screening method used to identify virus–host interactions; particularly useful for genes that require induction or are not ectopically expressed in the model cell line used [134]. cDNA libraries can range in size [135], for example, Nguyen *et. al* used a cDNA library of 15000 genes to identify proviral host factors for HIV-IIIb infection in HIV-permissive HeLaCD4βgal cells [136]. Subsequently, 315 HFs that increased infection were identified, including mixed lineage kinase 3 (MLK3).

Quantitative proteomics has also been used to identify antiviral proteins. Stable isotope labelling by amino acids in cell culture (SILAC), alongside other labelling methods such as tandem mass tagging (TMT) or label-free setups, can be used to compare virus and mock infected cells and identify differences in protein abundance between the two populations [137]. Nightingale *et al*. used a multiplexed proteomics approach to identify HCMV restriction factors, including helicase-like transcription factor (HLTF). Proteins rescued by proteasomal and lysosomal inhibitors upon HCMV infection, compared with mock infected cells, were identified using TMT peptide labelling and mass spectrometry. Hits were then further investigated by pSILAC to look at the rate of protein turnover in HCMV and mock infected cells [138].

## Future perspectives

Each of the screening techniques discussed has its place in investigating virus–host interactions both in the identification of host dependency factors and antiviral factors. As these techniques are further developed, as already observed for ISG overexpression libraries and CRISPR/Cas9 library design, the power of these techniques will also increase.

A consequence of specific ISGs remaining unknown for most viruses is the resulting difficulty in investigating other components of the antiviral response. An exception to this is PIV5, where IFIT1 is known to be the primary restriction factor [139]. Recently, Holthaus *et al*. developed an infection model using this information to determine that PIV5 virus resistance is a result of direct antiviral activity [114]. Therefore, a greater understanding of the antiviral response and the specific restriction factors involved, through high-throughput genetic screening, may allow investigation of the wider IFN response.

Genetic screens have also been developed to study pathogens of agricultural significance. Tan *et al*. have developed a bovine CRISPR/Cas9 knockout library, btCRISPRko.v1, consisting of 96 000 sgRNAs to identify genes involved in virus–host interactions. The btCRISPRko.v1 library has subsequently been used identify cellular factors involved in replication of bovine herpes virus type 1 (BHV-1), a virus that results in severe economic burden in the cattle industry, and resulted in the identification of over 150 proteins. The development of a bovine screening library will enable the investigation of virus–host interactions upon infection with other cattle pathogens [140]. Furthering our knowledge of not only bovine but other livestock diseases may help provide food security alongside aiding our understanding of zoonotic transmission as a barrier to zoonotic infection are the differences in host IFN response between species.

Currently, validation of RNAi screening results with replicative CRISPR/Cas9 screening has been marginal and those that have been performed have seen minimal overlap in results. In future, it may be that the combination of various screening techniques to validate hits will enable greater overlap between studies of the same virus. However, problems are still associated with these screens, including the standardisation of screening results. As the number of published data sets from screening, in any form, increases, a more comprehensive picture of virus–host interactions can be established. As more screens are performed, it is likely that standardisation between data sets will improve, allowing for comparison between screens and the identification of common hits. This will enable greater confidence in hits and provide evidence for therapeutic targets. Additionally, increased comparative screens between viruses may result in hits that can be exploited as broad therapeutic targets. ISG-specific libraries are now available for all three screening techniques discussed [23, 63, 87] enabling targeted screening of antiviral restriction factors. Targeted validation screening of antiviral factors, rather than performing a genome wide screen, is therefore possible and greater overlap of hits may be observed.

The use of genetic screening to identify not only antiviral factors, but host-dependency factors, undeniably has its benefits. Without a sufficient number of hits being identified under the same experimental conditions, clustering analysis to this level would not be feasible. It is these analyses that provide greater insight than single-gene studies can provide into virus–host interactions. Similarly, the level of redundancy of the IFN response, identified through high-throughput screening itself, provides reasons for the need for these methods. Despite the caveats regarding these methods, they are a powerful tool for investigating virus–host interactions.
